# Moderate to Severe Obstructive Sleep Apnea Is a Risk Factor for Severe COVID‐19—A Nationwide Cohort Study

**DOI:** 10.1111/jsr.70082

**Published:** 2025-05-05

**Authors:** Mirjam Ljunggren, Andreas Palm, Magnus Ekström, Josefin Sundh, Ludger Grote, Huiqi Li, Fredrik Nyberg, Össur Ingi Emilsson

**Affiliations:** ^1^ Department of Medical Sciences, Respiratory, Allergy and Sleep Research Uppsala University Uppsala Sweden; ^2^ Faculty of Medicine, Department of Clinical Sciences Lund, Respiratory Medicine, Allergology and Palliative Medicine Lund University Lund Sweden; ^3^ Department of Respiratory Medicine, Faculty of Medicine and Health Örebro University Örebro Sweden; ^4^ Centre for Sleep and Wakefulness Disorders, Institute of Medicine, Sahlgrenska Academy Gothenburg University Gothenburg Sweden; ^5^ Pulmonary Department Sahlgrenska University Hospital Gothenburg Sweden; ^6^ School of Public Health and Community Medicine, Institute of Medicine, Sahlgrenska Academy University of Gothenburg Gothenburg Sweden; ^7^ Faculty of Medicine University of Iceland Reykjavik Iceland

**Keywords:** CPAP, oxygen desaturation index, SARS‐CoV2, sleep disordered breathing

## Abstract

The impact of obstructive sleep apnea (OSA) and positive airway pressure (PAP) treatment on COVID‐19 severity is unclear. In this population‐based, nationwide study using multi‐register data, we aimed to assess if OSA is a risk factor for COVID‐19 severity and how adherence to PAP treatment and clinical characteristics affect the risk. Swedish residents with COVID‐19 infection January 2020–May 2022 were included. An exposed group of OSA (starting PAP treatment 2015–2019) was identified. COVID‐19 severity outcome was defined as mild (non‐hospitalised), severe (hospitalised) or critical (intensive care or death). Covariates included comorbidities and sociodemographics. Conditional odds ratios (COR) with 95% confidence intervals (95% CI) were estimated using multinomial logistic regression. Among 8,894,162 individuals in Sweden, 1,932,081 (21.7%) had registered COVID‐19 January 2020–May 2022. OSA was identified in 11,407 (0.6%) and was associated with an increased risk of severe (COR 1.34; 95% CI 1.25–1.43) and critical (1.25; 1.11–1.42) COVID‐19 after adjustment for age, sex, education and comorbidities. Stratified by PAP adherence, age and COVID‐19 wave, OSA was a risk factor for more severe COVID‐19 in PAP‐adherent and non‐adherent individuals, in people aged 40–60 but not > 60 years and not after June 2021. OSA severity, assessed with the oxygen desaturation index (ODI), was independently associated with COVID‐19 severity, with the highest risks for severe (1.23; 1.01–1.52) and critical (1.76; 1.17–2.63) COVID‐19 observed in ODI ≥ 30 (vs. ODI < 15). We conclude that patients with moderate to severe OSA have an increased risk of severe COVID‐19, also when PAP‐treated, with an independent dose–response relationship between the severity of intermittent hypoxia and COVID‐19 severity.

**Abbreviations**
ACE2: angiotensin converting enzyme 2
AHI: apnea‐hypopnea index
ATC: Anatomical Therapeutic Chemical
BMI: body mass index
CI: confidence interval
COR: conditional odds ratios
ICD10: International Classification of Diseases, Tenth Revision
LISA: the Swedish Longitudinal Integrated Database for Health Insurance and Labour Market Studies
ODI: oxygen desaturation index
OSA: obstructive sleep apnea
PAP: positive airway pressure
RAAS: renin‐angiotensin‐aldosterone system
SCIFI‐PEARL: Swedish Covid‐19 Investigation for Future Insights—A Population Epidemiology Approach Using Register Linkage
SmiNet: the Swedish national surveillance system for reportable infectious diseases
Swedevox: the Swedish National Register of Respiratory Failure

## 1 Introduction

The risk factors for severe COVID‐19 are numerous, including higher age, male sex, obesity and hypertension. As these factors are also strongly associated with obstructive sleep apnea (OSA), early studies found, as expected, OSA to be associated with severe COVID‐19, although they generally did not adjust for these common risk factors. In later studies, where possible confounding factors have been addressed thoroughly, OSA seems still to be a moderate risk factor for severe COVID‐19 (Grote 2022; Quan et al. 2023).

The possible mechanisms through which OSA may cause more severe COVID‐19 include upregulated renin‐angiotensin‐aldosterone system (RAAS) (Jin and Wei 2016), as the SARS‐CoV2 virus uses angiotensin converting enzyme 2 (ACE2) as an entry receptor (Beacon et al. 2021), as well as upregulated systemic inflammation (Ljunggren et al. 2024).

Upregulation of the RAAS system and systemic inflammation are known to be diminished when OSA is treated with positive airway pressure (PAP) treatment (Xie et al. 2013; Nicholl et al. 2021). Therefore, it is intriguing to explore if PAP treatment mitigates the risk for severe COVID‐19 among OSA patients. A few studies have tried to address this question as a secondary analysis, albeit they were underpowered and/or lacked data on PAP adherence (Quan et al. 2023; Rognvaldsson et al. 2022; Sampol et al. 2022; Nassi‐Liberman et al. 2025; Kendzerska et al. 2023). The results of these studies were discordant, with three studies reporting PAP‐treated OSA to be associated with a worse outcome from COVID‐19 (Rognvaldsson et al. 2022; Nassi‐Liberman et al. 2025; Kendzerska et al. 2023), the other two did not (Quan et al. 2023; Sampol et al. 2022). It is therefore still unclear if patients with PAP‐treated OSA have an elevated risk for severe COVID‐19.

Although the overall risk of severe COVID‐19 has decreased over time due to changing viral virulence, natural immunity, vaccination and treatment strategies, there is still a risk for severe disease in individual cases, with age as the strongest risk factor and an increasing risk with an increasing number of underlying risk factors (World Health Organization 2025; CDC 2025). Studies performed in early waves of the pandemic, or without stratification by wave, might not accurately reflect the impact of risk factors after the establishment of standardised treatment and start of mass vaccinations.

Our aim was to assess if OSA is a risk factor for COVID‐19 severity, and if the risk varies by age, COVID‐19 waves, OSA severity and PAP treatment time and compliance, using register data for the entire Swedish population. We hypothesised that OSA, in particular when associated with significant hypoxic burden, was an independent risk factor for a more severe COVID‐19.

## 2 Methods

### 2.1 Study Population With COVID‐19 and Severity of Outcome

In this population‐based, nationwide study, based on data from the Swedish Covid‐19 Investigation for Future Insights—A Population Epidemiology Approach Using Register Linkage (SCIFI‐PEARL) study (Nyberg et al. 2021) currently extended to a full population design, we included all people in Sweden aged ≥ 18 years on 1 January 2020 with registered COVID‐19 from January 2020 until May 2022. COVID‐19 was defined as either having a positive SARS‐CoV‐2 PCR test in the national surveillance system for reportable infectious diseases (SmiNet) (SmiNet 2021) or having a clinical diagnosis of COVID‐19 (International Classification of Diseases, Tenth Revision (ICD10)‐code U07.1, U07.2) in the National Patient Register (Ludvigsson et al. 2011) or the Cause‐of‐Death register (Brooke et al. 2017) from January 2020 until May 2022, and the index date for COVID‐19 was set to be 2 days before the earliest indication of any of these. COVID‐19 severity assessed as an outcome was graded in three grades: (1) mild (non‐hospitalised), (2) severe (hospitalised; not admitted to intensive care unit or dead from COVID‐19), (3) critical (admitted to intensive care unit; or death from COVID‐19).

### 2.2 Registered OSA as Exposed Group

Individuals with OSA in the study population were identified using data from the Swedish National Register of Respiratory Failure (Swedevox) on patients starting PAP treatment for OSA in 2015–2019 (Ekstrom et al. 2021). This registered OSA group constitutes predominantly patients considered to suffer from clinically relevant moderate to severe OSA. PAP is by far the most prevalent first treatment choice in Sweden. Compared to other treatment modalities (Mandibular Advancement Device or upper airway surgery) patients started on PAP have a more severe OSA with more nocturnal hypoxic load compared to the remaining OSA group in Sweden (Grote et al. 2024).

OSA severity was assessed with two different measurements: (1) Apnea‐hypopnea index (AHI), categorised using standard clinical cutpoints as mild (< 15 events/h), moderate (15–< 30 events/h) or severe (≥ 30 events/h) and (2) oxygen desaturation index (ODI) categorised using the same cutpoints (*Sleep* 1999). The time since start of PAP until the end of 2019 was calculated, and time on PAP treatment up to 2019 was categorised as < 2 years, 2–4 years or > 4 years.

Swedevox data from 1‐year follow‐up after start of PAP treatment, only available in a subsample, included information on the PAP use time from the PAP machines' data log. Adherence was defined as mean PAP usage ≥ 4 h/night during follow‐up and non‐adherence as usage < 4 h/night or not using PAP (Palm et al. 2021).

### 2.3 Covariates

Prevalent comorbidities at baseline (1 January 2020), associated with increased risk of severe COVID‐19 and OSA, were identified from National Patient Register data (Ludvigsson et al. 2011) from specialist in‐ and outpatient care 2015–2019 and Prescribed Drug Register data (Wallerstedt et al. 2016) 2018–2019.

Diabetes was defined as any dispensations of prescribed diabetes medications (Anatomical Therapeutic Chemical [ATC] codes A10) from the National Prescribed Drug Register. Cardiovascular disease was identified using National Patient Register data on hypertension (ICD‐code I10‐I15), ischaemic heart disease (ICD‐10 codes I20—I25, Z951, Z955) and heart failure (ICD‐10 codes I50). Chronic lung diseases were captured by regular inhaler use (ATC‐codes R03A, R03BA, R03BB, R03C, R03D) based on Prescribed Drug Register data and by Patient Register data on severe respiratory disease (including emphysema, chronic obstructive pulmonary disease, interstitial lung diseases, chronic respiratory failure and cystic fibrosis captured by ICD‐10 codes J43, J44.8, J44.9, J84, J96.1, J96.9, E84). Severe chronic kidney disease was defined as having a diagnosis of chronic renal disease stage 4–5 or dialysis (ICD‐10 codes N18.4, N18.5, Z99.2) in the National Patient Register.

Education level, based on data from the Swedish Longitudinal Integrated Database for Health Insurance and Labour Market Studies (LISA) (Ludvigsson et al. 2019) was defined as the highest education level up until the end of 2019 and was categorised into three(1) Primary school, (2) Secondary school, (3) University. The healthcare region the participants were living in at the index date for the COVID‐19 infection was also registered.

Data on body mass index (BMI) was taken from any available register, and if multiple values were available, the most recent value before 1 January 2020 was used. For individuals with registered OSA, BMI data was available from the Swedevox register (Ekstrom et al. 2021). BMI was categorised as underweight (< 18.4), normal weight (18.5–25), overweight (25.1–30) or obesity (> 30).

### 2.4 Statistical Analysis

In the study population, multinomial logistic regression models were created to analyse the associations between registered OSA (compared to the rest of the COVID‐19 population) as an independent variable and COVID‐19 severity (with mild disease as reference category) as the dependent variable. Conditional odds ratios (COR) with 95% confidence intervals (95% CI) were estimated (Gould 2000). The models were adjusted for the covariates age, sex, education, diabetes, hypertension, ischemic heart disease, heart failure, regular inhaler use, severe respiratory disease, severe chronic kidney disease and healthcare region. The analyses were first performed in the entire COVID‐19 infected cohort and then stratified by age (40–60 years, > 60 years) and COVID‐19 waves, with individuals categorised based on their COVID‐19 index date, using the following time frames: (1) Feb 2020–Jan 2021; (2) Feb 2021–Jun 2021; (3) Jul 2021–May 2022. The time frames were defined based on definitions of pandemic phases by the National Board of Health and Welfare, with the first two phases merged (Socialstyrelsen, n.d.). Since BMI data was only available in a very small subsample of the control group, adjustment for BMI was not possible in these analyses. The number of individuals with registered OSA aged < 40 years was low, and therefore this age group was excluded from the age‐stratified analysis.

The main analysis was then repeated, restricting the OSA group to the subset with 1‐year follow‐up data, where PAP‐treated OSA could be stratified into non‐adherent and adherent patients. The association of these PAP adherence groups to COVID‐19 severity (with mild COVID‐19 as reference category) was assessed using the COVID‐19 infected population without registered OSA as the reference. The model was adjusted for the same set of confounders as in the main analysis, and the same time frames were used. Individuals with registered OSA without 1‐year follow‐up data were excluded from this analysis.

Within the sup‐population of registered OSA patients with COVID‐19, associations between severity of OSA, as well as duration of treatment, and risk of severe COVID‐19 were analysed using multinomial logistic regression models. AHI (< 15 events/h, 15–< 30 events/h, ≥ 30 events/h), ODI (< 15 events/h, 15–< 30 events/h, ≥ 30 events/h) and time on PAP treatment (< 2 years, 2–4 years or > 4 years) were used as independent variables in separate models, thus comparing moderate and severe OSA and long time on treatment with mild OSA and short time on treatment, respectively. These models were adjusted for BMI in addition to the set of confounders used in the models in the entire cohort.

### 2.5 Ethical Considerations

The study was approved by the Swedish Ethical Review Authority (2020‐01800 with subsequent amendments), which waived individual informed consent due to the use of national registry data.

## 3 Results

Out of 8,894,162 individuals in Sweden aged ≥ 18 years, 1,932,081 individuals (21.7%) had a registered COVID‐19 during the study period and were included in the COVID‐19 infected study population. Registered OSA at baseline was identified in 11,407 (0.6%) of the COVID‐19 infected population. Compared to those without, COVID‐19 infected people with registered OSA were older, more often men, and had more cardiometabolic and respiratory comorbidities (Table 1). The median AHI in the registered OSA group was 30.0 events/h (interquartile range 18.0–48.0), indicating predominantly severe sleep‐disordered breathing in this group. The vast majority of people with registered OSA were either overweight (28.7%) or obese (63.9%).

**TABLE 1:** Baseline characteristics of the study population with COVID‐19 infection January 2020–May 2022, without and with registered OSA.

	General population	Registered OSA
	*N* = 1,920,674	*N* = 11,407
Male, *n* (%)	892,423 (46.5%)	8030 (70.4%)
Age, years	41.0 (30.0–53.0)	54.0 (46.0–62.0)
Diabetes, *n* (%)	79,414 (4.1%)	1785 (15.6%)
Hypertension, *n* (%)	107,501 (5.6%)	2793 (24.5%)
Ischaemic heart disease, *n* (%)	34,271 (1.8%)	816 (7.2%)
Heart failure, *n* (%)	20,039 (1.0%)	488 (4.3%)
Regular inhaler use, *n* (%)	154,853 (8.1%)	1941 (17.0%)
Severe respiratory disease, *n* (%)	13,897 (0.7%)	335 (2.9%)
Severe chronic kidney disease, *n* (%)	5141 (0.3%)	93 (0.8%)
Education level, *n* (%)		
Primary	248,005 (13.3%)	1770 (15.6%)
Secondary	832,474 (44.5%)	6012 (53.1%)
University	790,582 (42.3%)	3542 (31.3%)
COVID‐19 severity, *n* (%)		
Mild COVID‐19	1,826,041 (95.1%)	10,092 (88.5%)
Severe COVID‐19	70,000 (3.6%)	1019 (8.9%)
Critical COVID‐19	24,633 (1.3%)	296 (2.6%)
Diagnostic AHI, events/h		30.0 (18.0–48.0)
Diagnostic ODI, events/h		26.0 (16.0–45.0)
Duration of therapy, days		1408.0 (1008.0–1902.0)
BMI‐categories, *n* (%)		
Underweight		14 (0.1%)
Normal weight		786 (7.2%)
Overweight		3140 (28.7%)
Obesity		6986 (63.9%)

*Note*: Baseline characteristics on 1 January 2020 of the study population of adults in Sweden with COVID‐19 infection January 2020–May 2022, for individuals without and with registered OSA. Data presented as, *n* (%) or median (interquartile range).

Abbreviations: AHI = apnea hypopnea index, BMI = body mass index, ODI = oxygen desaturation index, OSA = obstructive sleep apnea.

**FIGURE 1:**
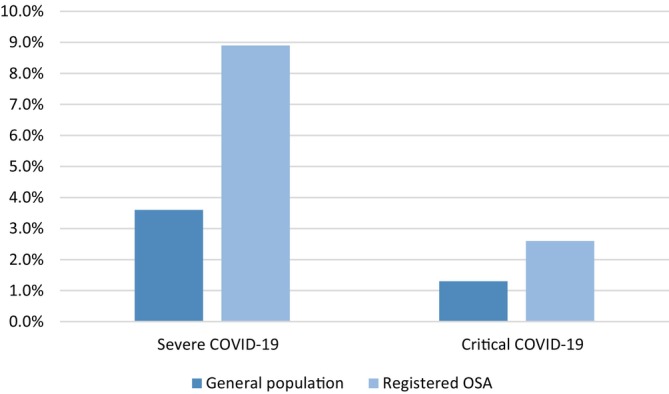
COVID‐19 severity in individuals without and with registered OSA. Proportion of severe and critical COVID‐19 among adults in Sweden with COVID‐19 infection January 2020–May 2022, for individuals without and with registered OSA. OSA = obstructive sleep apnea.

COVID‐19 was more commonly severe or critical in OSA than in the population without registered OSA (8.9% vs. 3.6% and 2.6% vs. 1.3%, respectively) (Figure 1) and OSA was associated with an increased conditional odds ratio for severe and critical COVID‐19 after adjustment for age, sex, educational level and comorbidities (Table 2).

**TABLE 2:** Associations between registered OSA and COVID‐19 severity in the entire COVID‐19 infected cohort and stratified by age and wave.

	Severe COVID‐19 COR (95% CI)	Critical COVID‐19 COR (95% CI)
Entire cohort, *n* = 1,882,385		
General population	Ref	Ref
Registered OSA	**1.34 (1.25–1.43)**	**1.25 (1.11–1.42)**
Stratified by age		
Age 40–60 years, *n* = 710,513		
General population	Ref	Ref
Registered OSA	**1.46 (1.32–1.62)**	**1.34 (1.06–1.69)**
Age > 60 years, *n* = 250,934		
General population	Ref	Ref
Registered OSA	1.06 (0.96–1.17)	1.07 (0.92–1.24)
Stratified by wave		
Wave 1 (Feb 2020–Jan 2021), *n* = 482,684		
General population	Ref	Ref
Registered OSA	**1.33 (1.20–1.47)**	**1.18 (1.00–1.39)**
Wave 2 (Feb 2021–Jun 2021), *n* = 393,724		
General population	Ref	Ref
Registered OSA	**1.48 (1.29–1.69)**	**1.55 (1.22–1.98)**
Wave 3 (Jul 2021–May 2022), *n* = 1,005,875		
General population	Ref	Ref
Registered OSA	1.12 (0.96–1.29)	1.14 (0.81–1.61)

*Note*: Associations between registered OSA (regardless of severity) and COVID‐19 severity compared with the general population without registered OSA, amongst adults in Sweden with COVID‐19 infection January 2020–May 2022. Analysed in the entire COVID‐19 infected cohort and stratified by age and wave. Results from multinomial logistic regression analysis in COVID‐19 infected, with mild COVID‐19 as reference, adjusted for age, sex, education, diabetes, hypertension, ischemic heart disease, heart failure, regular inhaler use, severe respiratory disease, severe chronic kidney disease, and healthcare region. Bold font indicates statistical significance.

Abbreviations: CI = confidence interval, COR = conditional odds ratio, OSA = obstructive sleep apnea.

Several secondary analyses were performed. First, registered OSA was associated with an increased risk for severe and critical COVID‐19 in individuals aged > 40 to ≤ 60 years, but not in those > 60 years (Table 2). Second, when stratified by wave, OSA was a significant risk factor for severe and critical COVID‐19 in the first and second COVID‐19 waves, but it was non‐significantly raised in the third (Table 2). Third, data on PAP compliance was only available in 28.4% of the PAP‐treated OSA patients. In this subsample, OSA was a risk factor for severe and critical COVID‐19 in both adherent and non‐adherent OSA patients compared with the COVID‐19‐infected population without registered OSA (Table 3). Fourth, duration of PAP treatment was associated with COVID‐19 risk. Of people with registered OSA, 9.0%, 44.3% and 46.7% had been on PAP therapy for < 2 years, 2–4 years and > 4 years, respectively. Compared to those with a short duration of therapy, the risk of critical COVID‐19 was lower in those with a longer duration of therapy (Table 4). Also, when time on PAP therapy was analysed as a continuous variable, longer duration of therapy associated with lower risk of critical COVID‐19 (COR per year: 0.91; 0.85–0.98).

**TABLE 3:** Associations between PAP‐treated OSA and COVID‐19 severity.

	Severe COVID‐19 COR (95% CI)	Critical COVID‐19 COR (95% CI)
General population	Ref.	Ref.
PAP treated OSA		
Non‐adherent	**1.50 (1.19–1.90)**	**1.53 (1.03–2.26)**
Adherent	**1.33 (1.14–1.55)**	**1.61 (1.27–2.05)**

*Note*: Associations between PAP‐treated OSA and COVID‐19 severity in non‐adherent and adherent OSA patients, compared with the general population without registered OSA, among adults in Sweden with COVID‐19 infection January 2020–May 2022. Results from multinomial logistic regression analysis in COVID‐19 infected (*n* = 1,874,278), with mild COVID‐19 as reference, adjusted for age, sex, education, diabetes, hypertension, ischemic heart disease, heart failure, regular inhaler use, severe respiratory disease, severe chronic kidney disease, and healthcare region. Bold font indicates statistical significance.

Abbreviations: CI = confidence interval, COR = conditional odds ratio, OSA = obstructive sleep apnea, PAP = positive airway pressure.

**TABLE 4:** Association between time on PAP treatment for OSA and COVID‐19 severity.

	Severe COVID‐19 COR (95% CI)	Critical COVID‐19 COR (95% CI)
Time on PAP treatment		
< 2	Ref	Ref
2–4 years	0.81 (0.64–1.04)	**0.46 (0.32–0.66)**
≥ 4 years	0.87 (0.68–1.11)	**0.50 (0.35–0.71)**

*Note*: Association between time on PAP treatment up to 2019 for OSA and COVID‐19 severity, among registered OSA patients in Sweden with COVID‐19 infection January 2020 until May 2022. Results from multinomial logistic regression analysis in PAP‐treated OSA patients with COVID‐19 (*n* = 10,849), with mild COVID‐19 as reference, adjusted for BMI, age, sex, diabetes, hypertension, educational level, ischemic heart disease, heart failure, regular inhaler use, severe respiratory disease, severe chronic kidney disease and healthcare region. Bold font indicates statistical significance.

Abbreviations: CI = confidence interval, COR = conditional odds ratio, OSA = obstructive sleep apnea, PAP = positive airway pressure.

**FIGURE 2:**
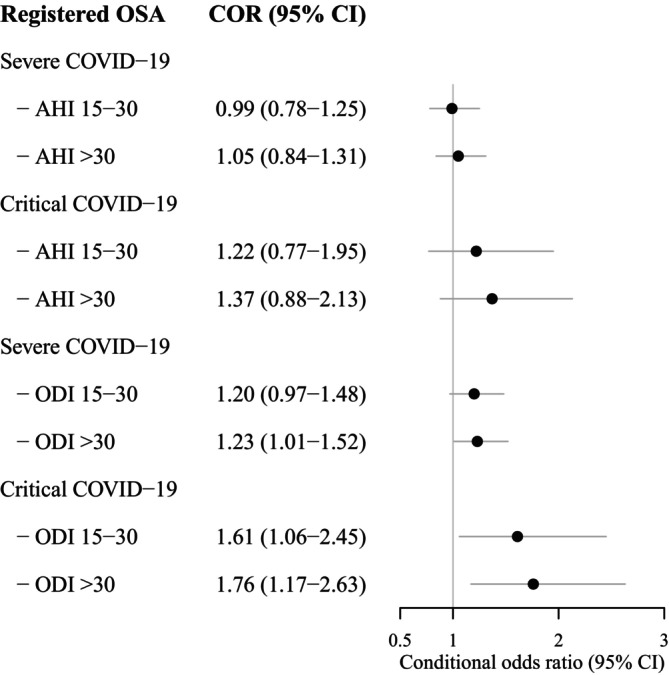
OSA severity as a risk factor for severe and critical COVID‐19. Results from adjusted^a^ multinomial logistic regression analysis in registered OSA patients with COVID‐19 in Sweden January 2020–May 2022 (*n* = 10,687), with mild COVID‐19 as reference. AHI and ODI (events/h) in separate models. ^a^Adjusted for BMI, age, sex, diabetes, hypertension, educational level, ischaemic heart disease, heart failure, regular inhaler use, severe respiratory disease, severe chronic kidney disease, and healthcare region. AHI = apnea hypopnea index, COR = conditional odds ratio, ODI = oxygen desaturation index, OSA = obstructive sleep apnea.

Finally, in the OSA group, those with more severe intermittent hypoxia, assessed with ODI, had an increased risk for severe and critical COVID‐19 compared with those with mild OSA with ODI < 15 events/h. In the final model fully adjusted for BMI, age, sex, educational level and comorbidities, the highest risks were observed in those with ODI ≥ 30 events/h compared with ODI < 15 events/h (Figure 2). Conversely, OSA severity assessed with AHI was not significantly associated with more severe COVID‐19.

## 4 Discussion

In this nationwide study, COVID‐19 was more often severe or critical in people with registered OSA, both in patients adherent and in those non‐adherent to PAP treatment. There was an independent dose–response relationship between the severity of intermittent hypoxia assessed with ODI and worse COVID‐19 severity. The increased risk of more severe COVID‐19 for OSA was seen in middle‐aged people, but not in older people. Also, the elevated risk of a more severe COVID‐19 in registered OSA diminished during the last wave of infections analysed (after June 2021), when mass vaccination had begun.

This is, to our knowledge, the first study to report a dose–response relationship between OSA severity assessed with ODI and risk for more severe COVID‐19. The results are, however, in line with meta‐analyses of previous studies reporting OSA (vs. no OSA) to be a risk factor for hospitalisation (Bellou et al. 2022) and death (Hu et al. 2022) in patients with COVID‐19.

Regarding PAP‐treated OSA specifically and COVID‐19 severity, previous studies are scarce and conflicting, and most are considerably smaller than the current study. Sampol et al., in a cohort of patients admitted to hospital for COVID‐19, with data on PAP‐treated OSA and adherence from an electronic database, found no association between PAP‐treated OSA (*n* = 81) and risk for severe respiratory failure or death (Sampol et al. 2022). Kendzerska et al. (2023) on the other hand, in a large register‐based study, reported an increased risk of severe COVID‐19 in patients with PAP therapy prescribed in the 5 years before the pandemic. However, they did not have data on PAP adherence, time on therapy or OSA severity. Rognvaldsson et al. (2022) also reported an increased risk of severe COVID‐19, defined as hospitalisation or death, in PAP‐treated OSA (prescribed PAP and had not returned the PAP device, *n* = 90) compared to COVID‐19 without OSA. In line with our finding that the risk of critical COVID‐19 was lower in those with PAP treatment for more than 2 years compared with those with a shorter duration of PAP treatment, Rognvaldsson et al. reported a trend towards lower risk for severe COVID‐19 in OSA with long time on PAP treatment. The reason for this may be related to long‐term effects on the immune system and low‐grade inflammation, as discussed further below.

The finding of increased risk of worse COVID‐19 in those aged 40–60 years, but not among older people, is in accordance with studies investigating cardiovascular endpoints in OSA, where the association between OSA and risk of cardiovascular disease is stronger at a younger age (Gottlieb et al. 2010; Haas et al. 2005). Another explanation might be that age is known to be the strongest risk factor for adverse COVID‐19 outcomes (CDC 2025) and the contribution of OSA to overall risk becomes less prominent. We also found an increased risk in the beginning of the pandemic, but not after June 2021. At this time point over ¾ of the adult Swedish population had received at least one dose of COVID‐19 vaccine (Ljung et al. 2021). Few other studies have investigated the risk of severe COVID‐19 in PAP‐treated OSA stratified by wave; there are, however, studies reporting an attenuation of the association between OSA and COVID‐19 severity with vaccination (Quan et al. 2023; Strausz et al. 2024). Vaccination was accompanied by a substantial reduction of severe COVID‐19 and also of adverse cardiovascular outcomes in the Swedish population, and our data further support that vaccination is highly effective and should be encouraged among individuals with comorbid moderate to severe OSA (Xu et al. 2025).

Our study was not designed to study potential mechanisms through which OSA may cause a more severe COVID‐19 infection. However, in the current study, intermittent hypoxia during sleep was strongly associated with COVID‐19 severity in a dose response manner. Interestingly, upregulation of the RAAS system and increased levels of angiotensin converting enzyme 2 have been linked to intermittent hypoxia as a key pathomechanism of OSA (Jin and Wei 2016; Ljunggren et al. 2024). In the context of COVID‐19, upregulated angiotensin receptors may have been involved in the elevated susceptibility for infection as they serve as entry receptors of the SARS‐CoV2 virus into respiratory epithelial cells (Beacon et al. 2021). Furthermore, apnea‐associated intermittent hypoxia in OSA is known to be associated with increased inflammation (Svensson et al. 2012). It is possible that the pre‐existing low‐grade inflammation in moderate to severe OSA has increased the risk for hyperactivation of the inflammatory cascade described as cytokine storm in more severe COVID‐19. Interestingly, the risk for severe COVID‐19 was lower in those OSA patients using PAP treatment for more than 2 years compared to those with shorter duration of PAP treatment, suggesting that the risk associated with OSA may take some time on treatment to mitigate.

Strengths of this study include the very large cohort with a nationwide design, including all laboratory‐confirmed SARS‐CoV‐2 infections and COVID‐19 diagnoses in Sweden, combined with valid data on OSA, PAP‐treatment, comorbidities, hospitalisations and mortality from Swedish high‐quality registers. With the current design based on rigorously monitored and validated national data, we do not expect a specific bias towards COVID‐19 severity classification in the PAP‐treated OSA group. The long study period provides additional strength, enabling analysis of variations between different COVID‐19 waves. Thereby, the findings of our study provide a high degree of generalisability for populations with comparable sleep apnea care models and healthcare settings. However, a few limitations need to be addressed as well. First, we did not have data on BMI for the entire study population, only for the subgroup with registered OSA. The results presented in Table 2, with OSA as a risk factor for severe COVID‐19, may therefore to some extent be explained by obesity. However, studies have shown a strong collinearity between the number of comorbidities and obesity, and therefore our rigorous model adjustment for comorbidities may in part have compensated for the lack of information about BMI. Furthermore, we identified a clear association between OSA severity (assessed with ODI) and COVID‐19 severity in BMI‐adjusted models, further suggesting an independent role of OSA‐related intermittent hypoxia for COVID‐19 severity and risk. Second, there are some individuals with OSA also in the general population group without PAP treatment, causing an underestimation of the effect of moderate–severe OSA on COVID‐19 severity. However, the average severity of OSA in this general population group, compared with the registered OSA group, is so mild that it is not likely to have any substantial effect on the results. In a Swedish general population sample aged 50–64 years, the mean AHI was 7.5 events/h and mean ODI 5.2 events/h (Delshad et al. 2024).

Taken together, moderate to severe OSA appears to increase the risk of more severe COVID‐19, especially in patients with severe intermittent hypoxia, and this risk may be mitigated in patients with long‐term PAP treatment. However, the increased risk was confined to middle‐aged patients with OSA and was stronger at the beginning of the pandemic, suggesting that in fully vaccinated and older individuals with long‐term PAP treatment, OSA should not be regarded as a risk factor of concern for more severe COVID‐19.

In conclusion, patients with moderate to severe OSA have an increased risk of more severe COVID‐19, also when PAP‐treated, with a dose–response relationship between the severity of intermittent hypoxia and COVID‐19 severity, independent of BMI, age, sex and comorbidities. However, OSA was not associated with an increased risk of more severe COVID‐19 in those aged > 60 years nor in the period after the start of mass vaccinations.

## Author Contributions

**Mirjam Ljunggren:** writing – original draft, formal analysis, methodology, conceptualization, writing – review and editing, visualization. **Andreas Palm:** conceptualization, methodology, writing – review and editing. **Magnus Ekström:** conceptualization, methodology, writing – review and editing. **Josefin Sundh:** conceptualization, methodology, writing – review and editing. **Ludger Grote:** conceptualization, methodology, writing – review and editing. **Huiqi Li:** conceptualization, data curation, methodology, project administration, writing – review and editing. **Fredrik Nyberg:** conceptualization, data curation, methodology, project administration, funding acquisition, writing – review and editing. **Össur Ingi Emilsson:** conceptualization, visualization, formal analysis, writing – original draft, writing – review and editing, methodology.

## Conflicts of Interest

All authors have seen and approved the manuscript. F.N. owns some AstraZeneca shares. The remaining authors declare no conflicts of interest.

## Data Availability

Research data are not shared.
